# Pathogenic mechanisms of post-acute sequelae of SARS-CoV-2 infection (PASC)

**DOI:** 10.7554/eLife.86002

**Published:** 2023-03-22

**Authors:** Zaki A Sherif, Christian R Gomez, Thomas J Connors, Timothy J Henrich, William Brian Reeves, RECOVER Mechanistic Pathway Task Force, Boris D Julg, Steven B Bradfute, K Coombs, C Kim, Pras Jagannathan, Christian Bime, Erin Burke Quinlan, Michael A Portman, Maria Laura Gennaro, Jalees Rehman, Benjamin K Chen, Sindhu Mohandas

**Affiliations:** Infectious Disease Division, Massachusetts General Hospital, Ragon Institute of MGH, MIT and HarvardCambridgeUnited States; Center for Global Health, Department of Internal Medicine, University of New Mexico Health Sciences CenterAlbuquerqueUnited States; NIH RECOVER Research Initiative: Patient representativeNew YorkUnited States; NIH RECOVER Research Initiative: Patient representativeNew YorkUnited States; Division of Infectious Diseases and Geographic Medicine, Department of Medicine, Stanford UniversityStandfordUnited States; Division of Pulmonary, Allergy, Critical Care & Sleep Medicine, Department of Medicine, University of Arizona College of MedicineTucsonUnited States; National Center for Complementary and Integrative Health, National Institutes of HealthBethesdaUnited States; Seattle Children’s Hospital, Division of Pediatric Cardiology, Department of Pediatrics, University of WashingtonSeattleUnited States; Public Health Research Institute and Department of Medicine, Rutgers New Jersey Medical SchoolNewarkUnited States; Department of Biochemistry and Molecular Genetics, University of Illinois, College of MedicineChicagoUnited States; Division of Infectious Diseases, Department of Medicine, Icahn School of Medicine at Mount SinaiNew YorkUnited States; Department of Pediatrics, Children’s Hospital Los Angeles, Keck School of Medicine, University of Southern CaliforniaLos AngelesUnited States; 1 Department of Biochemistry & Molecular Biology, Howard University College of Medicine Washington, District of Columbia United States; 2 Division of Lung Diseases, National Institutes of Health (NIH), National Heart, Lung and Blood Institute (NHLBI) Bethesda United States; 3 Department of Pediatrics, Division of Critical Care, Columbia University Vagelos College of Physicians and Surgeons and New York - Presbyterian Morgan Stanley Children's Hospital New York United States; 4 Division of Experimental Medicine, University of California San Francisco United States; 5 Department of Medicine, Joe R. and Teresa Lozano Long School of Medicine, University of Texas San Antonio United States; Maine Medical Center Research Institute United States; Icahn School of Medicine at Mount Sinai United States

**Keywords:** angiotensin-converting enzyme, PASC, pathobiological mechanisms, pathophysiological mechanisms, post-viral syndromes, Long COVID, tissue damage

## Abstract

COVID-19, with persistent and new onset of symptoms such as fatigue, post-exertional malaise, and cognitive dysfunction that last for months and impact everyday functioning, is referred to as Long COVID under the general category of post-acute sequelae of SARS-CoV-2 infection (PASC). PASC is highly heterogenous and may be associated with multisystem tissue damage/dysfunction including acute encephalitis, cardiopulmonary syndromes, fibrosis, hepatobiliary damages, gastrointestinal dysregulation, myocardial infarction, neuromuscular syndromes, neuropsychiatric disorders, pulmonary damage, renal failure, stroke, and vascular endothelial dysregulation. A better understanding of the pathophysiologic mechanisms underlying PASC is essential to guide prevention and treatment. This review addresses potential mechanisms and hypotheses that connect SARS-CoV-2 infection to long-term health consequences. Comparisons between PASC and other virus-initiated chronic syndromes such as myalgic encephalomyelitis/chronic fatigue syndrome and postural orthostatic tachycardia syndrome will be addressed. Aligning symptoms with other chronic syndromes and identifying potentially regulated common underlining pathways may be necessary for understanding the true nature of PASC. The discussed contributors to PASC symptoms include sequelae from acute SARS-CoV-2 injury to one or more organs, persistent reservoirs of the replicating virus or its remnants in several tissues, re-activation of latent pathogens such as Epstein–Barr and herpes viruses in COVID-19 immune-dysregulated tissue environment, SARS-CoV-2 interactions with host microbiome/virome communities, clotting/coagulation dysregulation, dysfunctional brainstem/vagus nerve signaling, dysautonomia or autonomic dysfunction, ongoing activity of primed immune cells, and autoimmunity due to molecular mimicry between pathogen and host proteins. The individualized nature of PASC symptoms suggests that different therapeutic approaches may be required to best manage specific patients.

## Introduction

PASC co, which encompasses Long COVID or post-COVID syndrome, is a condition marked by the continuation of COVID-19 symptoms—or the emergence of new ones—4 or more weeks after infection with SARS-CoV-2 (http://www.CDC.gov), or those persistently symptomatic for >30 days from onset of infection (http://www.NIH.gov). Alternatively, the World Health Organization describes PASC (or Post COVID-19 condition) as occurring in individuals with a history of probable or confirmed SARS-CoV-2 infection, usually 3 months from the onset of COVID-19 with symptoms that last for at least 2 months and cannot be explained by an alternative diagnosis (http://www.WHO.int). The current PASC definition in the NIH RECOVER (REsearching COVID to Enhance Recovery) Initiative program is: ‘*ongoing, relapsing, or new symptoms, or other health effects occurring after the acute phase of SARS-CoV-2 infection (i.e., present four or more weeks after the acute infection*)’. The RECOVER Initiative is an NIH-funded project whose goal is to rapidly improve our understanding of and ability to predict, treat, and prevent PASC (post-acute sequelae of SARS-CoV-2), including Long COVID.

The symptoms of PASC whether in hospitalized or non-hospitalized patients are numerous and diverse and include shortness of breath, chest pain, cognitive impairment, and fever, among others (Michelen et al., 2021). Reports also reveal that there are wide and country-specific variations in the prevalence of PASC ranging from 3.3% in the UK, 13.9% in the USA, to 39% in Denmark and the Faroe Islands (Mantovani et al., 2022; Perlis et al., 2022; Petersen et al., 2022; Subramanian et al., 2022). The pathophysiologic mechanisms underlying PASC remain to be determined. However, the clinical features and epidemiology may provide insights into possible mechanisms. Although PASC may be a consequence of long-term organ damage attributable to acute-phase infection, it is postulated that specific mechanistic pathways exacerbate the initial illness adversely affecting many organs and resulting in the development of later symptoms. As such, this multifactorial condition displays over 200 symptoms and affects multiple tissues, organs, and biological systems (i.e., ears, eyes, head, nose, throat, kidney, brain, cardiopulmonary, endocrine, gastrointestinal (GI), musculoskeletal, neuropsychiatric, and systemic) to varying degrees. So far, immune dysregulation, autoimmunity, dysautonomia, endothelial dysfunction (ED), occult viral persistence, reactivation of pre-existing chronic viral infections as well as coagulopathies are some of the main underlying pathophysiological mechanisms described (Ahamed and Laurence, 2022; Al-Ramadan et al., 2021; Amenta et al., 2020; Camargo-Martínez et al., 2021; Carod-Artal, 2021; Choutka et al., 2022; Cortés-Telles et al., 2021; Doykov et al., 2020; Fernández-de-Las-Peñas et al., 2021; Gottschalk et al., 2023; Lee et al., 2021; Mantovani et al., 2022; McFarland et al., 2021; Oronsky et al., 2023; Silva Andrade et al., 2021; Song et al., 2021; Toniolo et al., 2021; Touyz et al., 2021; Townsend et al., 2021; Wirth and Scheibenbogen, 2021; Wong et al., 2021). Although PASC has been described as ‘a mysterious mix of symptoms with no clear pattern’, it bears similarities to other post-viral syndromes, and to myalgic encephalomyelitis/chronic fatigue syndrome (ME/CFS), certain phenotypic clusters are emerging in the literature.

This review will delineate the current knowledge about the underlying mechanisms and/or biological pathways responsible for tissue damage including pulmonary, cardiac, GI, neuronal, vascular, etc. associated with COVID-19 and the subsequent generation of symptoms. It will highlight the underlying causes of PASC and suggest ways to understand the clinical symptomatology for improved diagnostic and therapeutic procedures for the condition. We will also postulate on the pathophysiology and pathobiology of PASC by providing working hypotheses that could aid in further exploration of this multifaceted syndrome. This review will attempt to decipher the possible underlying tissue injury mechanisms for long-term residual illness post-SARS-CoV-2 infection.

## Insight into PASC disease pattern

Epidemiological studies in the UK and USA have found similar symptoms, but differing rates and durations of Long COVID (Groff et al., 2021). A UK study discovered that SARS-CoV-2 infection was linked to 62 symptoms lasting beyond 12 weeks, including anosmia, hair loss, sneezing, ejaculation difficulties, and reduced libido. The risk of Long COVID was higher among women, ethnic minorities, those with lower socio-economic status, smokers, obese individuals, and those with underlying health conditions, and increased with decreasing age (Subramanian et al., 2022). In contrast, Long COVID affects 7.3% of 3042 US survey respondents, equivalent to 18.8 million adults. Impacts on daily activities were severe for 25.3% of those with Long COVID and 28.9% had SARS-CoV-2 infection over a year ago. Women, those with comorbidities, and those unvaccinated or not boosted were at higher risk (Robertson et al., 2022). Studies from the USA and Europe have found that the prevalence of Long COVID is higher among women and those with multiple chronic conditions (Chen et al., 2022; Fernández-de-Las-Peñas et al., 2022; Robertson et al., 2022; Thompson et al., 2022). However, there is variation in risk factor data due to differences in study design, population studied, and definition of Long COVID. Often, studies lack a COVID-free control group, focus on hospitalized or healthcare-seeking populations, and have different definitions of Long COVID. These studies emphasize the high burden of Long COVID and the need for further research on its prevalence and risk factors.

## Clinical manifestations of PASC that require mechanistic understanding

The clinical manifestations of PASC vary widely. Common PASC symptoms include fatigue, dyspnea, cough, ‘brain fog’, headaches, sleep disturbance, impaired smell and taste, palpitations, chest pain, and orthostatic dizziness (Davis et al., 2021; Hernandez-Romieu et al., 2022; Ozonoff et al., 2022; Sneller et al., 2022; Sudre et al., 2021). Using machine-learning algorithms, Estiri et al. uncovered 33 phenotypes of PASC and it is likely that multiple different pathophysiologic mechanisms account for this heterogeneity (Estiri et al., 2021). Indeed, detailed multi-omic profiling of COVID-19 patients revealed unique associations between immunological signatures and specific PASC endotypes (Su et al., 2022). Future mechanistic studies will need to recognize the heterogeneity of the disorder. PASC is more common in individuals who suffered a severe vs mild acute COVID-19 illness (Hernandez-Romieu et al., 2022; LaVergne et al., 2021; Xie et al., 2021). In such patients, the illness shares features of post-intensive care syndrome (PICS) (Rawal et al., 2017) and findings observed in survivors of the SARS and MERS coronavirus epidemics (Greenhalgh et al., 2020; Tansey et al., 2007). Pathophysiologic mechanisms in this group may include the effects of SARS-CoV-2 infection, reactivation of other viruses such as the Epstein–Barr virus (EBV), autoimmunity, incomplete recovery from acute organ injury (e.g., lung, heart, kidney, brain, thrombosis), effects of treatments (e.g., mechanical ventilation, steroids), and exacerbation of pre-existing conditions (e.g., pre-diabetes, CKD, CHF). However, individuals with mild COVID-19 (Augustin et al., 2021; Estiri et al., 2021), and even individuals with no serologic evidence of prior SARS-CoV-2 infection (Krishna et al., 2022; Sneller et al., 2022), can experience PASC symptoms, suggesting that some features of PASC may represent responses to the stress of the pandemic, for example, social isolation, anxiety, loss of friends or family, work stress, and financial stress, in addition to the viral infection itself. The prevalence of PASC appears to be lower in the more recent COVID ‘waves’, especially with the Omicron variant, and lower in people who were vaccinated prior to contracting COVID-19 (Azzolini et al., 2022; Zisis et al., 2022), suggesting that SARS-CoV-2 variants, availability of newer treatments and immune responses may impact the pathogenesis of PASC. For example, recent research suggests that the use of nirmatrelvir/ritonavir during acute SARS-CoV-2 infection reduces the risk of PASC regardless of vaccination status or history of prior infection (Xie et al., 2022). Long COVID may also manifest as residual or new organ dysfunction which could be the result of acute organ injury during the COVID-19 infection, for example, ARDS, acute kidney injury, or myocarditis, or the result of chronic inflammation, ED, or autoimmunity triggered by the infection. The high incidence of new-onset type I diabetes after COVID-19 infection, for example, could be due to direct viral injury of pancreatic beta cells, induction of autoimmunity to beta cells or glucose-regulating pathways or decreases in peripheral insulin sensitivity associated with chronic inflammation that is exhibited in PASC patients (Barrett et al., 2022; Rubino et al., 2020). There are commonalities of clinical manifestations and symptoms between COVID-19 and Long COVID-19 that require detailed mechanistic understandings (Davis et al., 2021; Mehandru and Merad, 2022).

## Commonalities with ME/CFS and other post-infection syndromes

Many PASC features resemble (ME/CFS), which is also commonly preceded by viral infections, prompting some to ask whether PASC and ME/CFS are similar diseases (Bornstein et al., 2022; Tate et al., 2022). Fatigue, exertion intolerance, and post-exertional malaise are among the most frequent symptoms cited for PASC. These same symptoms manifesting in PASC patients had been designated as ‘core criteria’ of ME/CFS by the National Academy of Medicine in a 2015 report (https://batemanhornecenter.org/wp-content/uploads/filebase/providers/Recognizing-PVS-and-Tools-ECHO-5_3_2022-V2.pdf). It is estimated that about half of people with Long COVID will meet the criteria for ME/CFS, whether they are given that specific diagnosis or not. It is also important to note the multiple types of tissues and organs adversely affected by ME/CFS including cardiovascular problems, central nervous system (CNS), immune system disturbances, ion transport dysfunction, cell energy metabolism, GI dysfunction, cognitive impairment, myalgia, arthralgia, orthostatic intolerance, and chronic inflammation (Rasa et al., 2018), are also commonly reported in PASC patients. The trigger for COVID-19 is the SARS-CoV-2 infection. ME/CFS is a complex disease frequently triggered by an infection with EBV (the virus that causes mononucleosis) or parvovirus B19 even though various other viral and nonviral triggers have also been reported (Chu et al., 2019; Fluge et al., 2021; Jason et al., 2022; Sotzny et al., 2018). The evidence is clear that in COVID-19 and potentially in PASC patients, the initial SARS-CoV-2 infection resurrects latent viruses such as EBV (Fluge et al., 2021), which could trigger ME/CFS. Although EBV has been associated with post-viral fatigue in ME/CFS, the mechanisms leading to post-viral fatigue syndrome and Long COVID (PASC) are unresolved. The similarities raise two questions: *Do these two multi-symptomatic diseases share common molecular and pathophysiological pathways in their disruption of homeostasis post-infection? Although the triggers may be different, because of their overlapping symptoms, could there be a common pathobiological explanation?* Indeed, the lessons learned from three decades of studying ME/CFS should help with identification of a variety of conceptually distinct medical comorbid conditions found in PASC.

Postural orthostatic tachycardia syndrome (POTS), which can be seen after other viral infections has also been observed in PASC and may point toward autoimmune dysfunction of the autonomic system, including autoantibodies to acetylcholine and adrenergic receptors (Blitshteyn and Whitelaw, 2021; Fluge et al., 2021). However, a recent German study reports that PASC is not associated with autoantibodies (Schultheiss et al., 2022). Nevertheless, SARS-CoV-2 may be responsible for PASC symptoms by activating the host’s immune response and leading to long-term autoantibody production. Autoantibodies were isolated in acute COVID-19 patients by several research teams including Wang et al. who used REAP to screen a group of 194 SARS-CoV-2-positive COVID-19 patients for autoantibodies against 2770 extracellular proteins. Results showed that COVID-19 patients had a significant increase in autoantibody reactivity compared to uninfected controls (Jiang et al., 2023; Wang et al., 2021). It is currently not well understood what role autoantibodies may play in the pathophysiology of Long COVID or PASC. Evidence linking autoantibodies to Long COVID as an etiological factor is limited and further research is needed to fully understand their role in the development of PASC. Similarities between PASC symptoms and those of Mast Cell Activation Syndrome (MCAS) have also been noted (Akin, 2017; Blitshteyn and Whitelaw, 2021; Schieffer and Schieffer, 2022).

Longitudinal studies need to be conducted to determine the similarities and differences between PASC and ME/CFS and other post-infectious syndromes by frequently collecting biological samples (as it is being implemented by the RECOVER study—https://recovercovid.org), and up-to-date information on the presence of the infectious agent and the severity of various symptoms. Such studies should also include continual laboratory studies of the immune system, metabolism, gene structure and function, and transcriptome, as well as tests of cognition, sleep, and the functioning of the nervous system, heart, and cardiovascular system.

## Mechanisms of emerging PASC pathogeneses

Although the COVID-19 pandemic disproportionately affected patients who had comorbid diabetes mellitus, it is now clear from numerous studies across the globe that COVID-19 causes dysregulation of glucose homeostasis leading to new-onset hyperglycemia and type I diabetes (Genç et al., 2021) in patients with no previous risk factors for diabetes mellitus (Bode et al., 2020; Sathish et al., 2021c; Sathish and Chandrika Anton, 2021b; Tittel et al., 2020; Unsworth et al., 2020; Watson, 2022). New-onset COVID-induced type 1 or 2 diabetes has also been documented in children (Tittel et al., 2020; Trieu et al., 2021; Unsworth et al., 2020). This novel form of COVID-induced diabetes with anomalous glycemic parameters and heightened rates of DKA (diabetic ketoacidosis), occurs in severely ill patients with higher mortality rates and poorer outcomes compared to COVID-19 patients with pre-existing diabetes (Chandrashekhar Joshi and Pozzilli, 2022). The underlying pathobiological mechanism for this induced diabetes in COVID-19 patients has not been clearly delineated although it is suspected that SARS-CoV-2 could damage the beta cells of the pancreas and precipitate insulin resistance (Aluganti Narasimhulu and Singla, 2022). There are some plausible mechanisms that underlie this new diabetes onset. SARS-CoV-2 infection can damage beta cells or increase glucose levels via oxidative stress and inflammation; the virus can also bind the angiotensin-converting enzyme receptor 2 (ACE2), on acinar cells and cause tissue detriment and pulmonary fibrosis via increasing concentration of the fibrosis-promoting Angiotensin II (Morganstein et al., 2021) and hyperglycemia can inhibit lymphocyte proliferation to repair the tissue damage. The initial binding of the virus to ACE2 requires the presence of transmembrane serine protease 2 (TMPRSS2), a protein that is regulated by androgens. The ACE2 mRNA is also expressed in several endocrine glands, including the pancreas, ovaries, testes, and thyroid gland (Lazartigues et al., 2020). At postmortem, both follicular and parafollicular cells of the thyroid gland were extensively damaged in patients who died of COVID (Wei et al., 2007). These systems, which are physiologically regulated by ACE2 upon viral infection, induce acute cardiopulmonary failure, and coagulopathy. Furthermore, the overexpression of the ACE2 receptor can deregulate these systems and cause cardiovascular instability (renin–angiotensin system), acute inflammatory pulmonary edema (kinin–kallikrein system), and thromboembolism (coagulation system) (Sidarta-Oliveira et al., 2020). In fact, ACE2 gene therapy in gene knockout mice promoted better survival and function of the beta cells, as well as improved glucose homeostasis (Bindom et al., 2010). Additionally, a downregulation of the transcription factor REST (RE1-silencing transcription factor) observed in COVID-19 patients is linked to the altered gene expression of glucose and lipid metabolism involving apelin, myostatin myeloperoxidase (peptides and proteins important for the insulin signaling pathway) (He et al., 2021). COVID-19 patients also harbored upregulated short-chain fatty acids, propionic acid, and isobutyric acid, which are possibly linked to insulin resistance (He et al., 2021).

Stress is another factor that can be induced by a viral infection, which causes elevated glucose levels in subjects with diabetes via the secretion of glucocorticoids and catecholamines contributing to further impairment of the immune system (Wang et al., 2020). In addition, the incidence of DKA observed in COVID-induced diabetic patients was unusually high in the absence of autoantibodies suggesting that acute viral-induced pancreatic damage might have occurred (Li et al., 2020b; Vellanki and Umpierrez GE - Center ofDiabetes and Metabolism, Emory University School of Medicine, 2021). There are also a few reports of cases that have described autoantibody-negative insulin-dependent diabetes (Hollstein et al., 2020; Kuchay et al., 2020). Mechanistically, the consequence of stress caused by SARS-CoV-2 infection may be explained by the stimulation of a signaling pathway designated as the integrated stress response (ISR) that promotes the activation of a family of serine/threonine kinases that is, double-stranded RNA-dependent protein kinase (PKR) and PKR-like ER kinase (PERK), and the subsequent induction of serine phosphorylation of insulin receptor substrates (IRS) which leads to downregulation of the insulin signaling pathway. Both the viral components (RNA or proteins) of SARS-CoV-2 and ‘cytokine storm’, which activates a family of serine/threonine kinases linked to the ISR and is represented by pro-inflammatory cytokines such as IL-6 and tumor necrosis factor (TNF-α, can cause insulin resistance Santos et al., 2021). Moreover, insulin resistance can be caused by the downregulation of insulin receptors in skeletal muscle via virally induced interferon gamma (IFN-γ) (Sestan et al., 2018). This could be an additional contributor to the insulin resistance syndrome that has been described in COVID-induced diabetes patients.

There are also several other postulated mechanisms including molecular mimicry, that may explain the rise in autoimmune diabetes following COVID-19 infection. SARS-CoV-2 also downregulates ACE2, which degrades Angiotensin II. These mechanisms of unrestricted actions of Angiotensin II lead to deleterious effects including inflammatory activity by increasing the infiltration of macrophages and monocytes, reducing blood flow to pancreatic islets, and promoting the instability of the beta cells leading to glycemic dysregulation (Carlsson et al., 1998; Chee et al., 2020; Montezano et al., 2014). There is emerging evidence showing that the new-onset diabetes is also a feature of Long COVID (Sathish et al., 2021a) even though the precise mechanism might be multifaceted. It is essential to collect biospecimens to design mechanistic studies that might shed light on the underlying causes precipitating the various types of symptoms experienced by Long COVID sufferers and specifically to follow-up COVID-related diabetes patients by implementing longitudinal and prospective cohort studies to distinguish between COVID-induced diabetes from conventional diabetes. There is a digital registry that is specifically designed to establish the extent and characteristics of new-onset, COVID-19-related diabetes, and to investigate its pathogenesis and outcomes (http://covidiab.e-dendrite.com).

## Molecular mechanisms of respiratory PASC

The upper respiratory system is the primary site of the SARS-CoV-2 viral infection. In the scenario of an excessive inflammatory response, the consequence can be irreversible lung fibrosis and the compromise of respiratory effector function (Tanni et al., 2021). Fibrotic remodeling with characteristic findings consists of fibroblast proliferation, micro-honeycombing, and airspace obliteration, which were uncovered in pulmonary postmortem findings in a series of COVID-19 cases (Grosse et al., 2020). This suggested a link to respiratory distress syndrome (ARDS) as a long-term complication of COVID-19. In addition to ARDS, fibrotic remodeling resulting in respiratory symptoms can originate from pathologic conditions including dyspnea, and even from hospitalization, intensive care unit stays, use of high-flow oxygen support, and need for mechanical ventilation (Grillo et al., 2021). Additional mechanisms include epithelial and endothelial to mesenchymal transition and the ‘cytokine storm’. Prolonged exposure to supplemental oxygen is known to lead to increased oxidative stress and could maintain the inflammatory status favorable for activation of post-COVID-19 pulmonary fibrosis (George et al., 2020). A fraction of PASC patients present with fatigue, exercise limitation, persistent chest pain, and breathlessness. These symptoms may be caused in part by compromised cardiorespiratory function, lung vascular damage in microvessels, and hemodynamic complications such as resolved thrombus (Kosuge et al., 2012; Yaméogo et al., 2011). Fatigue and breathlessness may also be caused by chronic overstimulation of the ergoreflex, activated by exercise to centrally couple ventilation and cardiovascular function to exercise intensity (Sze et al., 2022). Exaggerated ergoreflex leads to excessive response relative to work performed, and to the sensation of breathlessness and fatigue. The hypercatabolic state, cytokine storm, and stimulation of the renin–angiotensin–aldosterone system by SARS-CoV-2 use of ACE2 to facilitate cell entry might cause a reduction in skeletal muscle mass and function, excessive ergoreflex, and sensations of fatigue and breathlessness (Medina-Enríquez et al., 2020). Further study of the mechanistic involvement and interactions between respiratory systems and extrapulmonary systems with reciprocal crosstalk and influence is needed.

Pulmonary dysfunctions are among the most destructive events associated with impaired immune responses caused by SARS-CoV-2 infection. The resulting cytokine storm activates defense events that stimulate biochemical pathways that eventually lead to the production of tissue injury markers and the collapse of lung tissue (Piazza et al., 2020; Sakr et al., 2020; Sidarta-Oliveira et al., 2020). These hematological changes form pulmonary thrombi and capillary microthrombi, which may represent thromboembolism instead of thrombosis in bone marrow emboli and septic pulmonary thromboembolism (Ackermann et al., 2020; D’Errico et al., 2020; Grosse et al., 2020; Khan et al., 2020). Transcriptomic analyses of coexpression data from human lung cells have shown that there are three physiological systems directly involved in the pathogenesis of COVID-19: (1) the kinin–kallikrein system; (2) the renin–system angiotensin; and (3) the coagulation system coexpressed with the ACE2 receptor in alveolar cells (Sidarta-Oliveira et al., 2020). These systems, which are physiologically regulated by ACE2 upon viral infection of the lungs, induce acute cardiovascular failure, coagulopathy, acute inflammatory pulmonary edema (kinin–kallikrein system), and thromboembolism (coagulation system) (Sidarta-Oliveira et al., 2020). Hyperinflammation induced by immunopathogenic, and biochemical mechanisms of SARS-CoV-2 suggests that cytokines and chemokines contribute to alveolar endothelial damage that promotes apoptosis of alveolar cells (pneumocyte type I) and degeneration of pulmonary pneumocyte type II alveolar cells (Hussman, 2020). These cell types are operationally connected via narrow junctions that control the transfer of ions, minerals, and fluid through the epithelium (Ackermann et al., 2020). SARS-CoV-2 infects ACE2-expressing pneumocyte type II cells, which act as epithelial immune cells and produce TNF, IL-6, IL-1β, and MCP-1.

**Figure 1.**
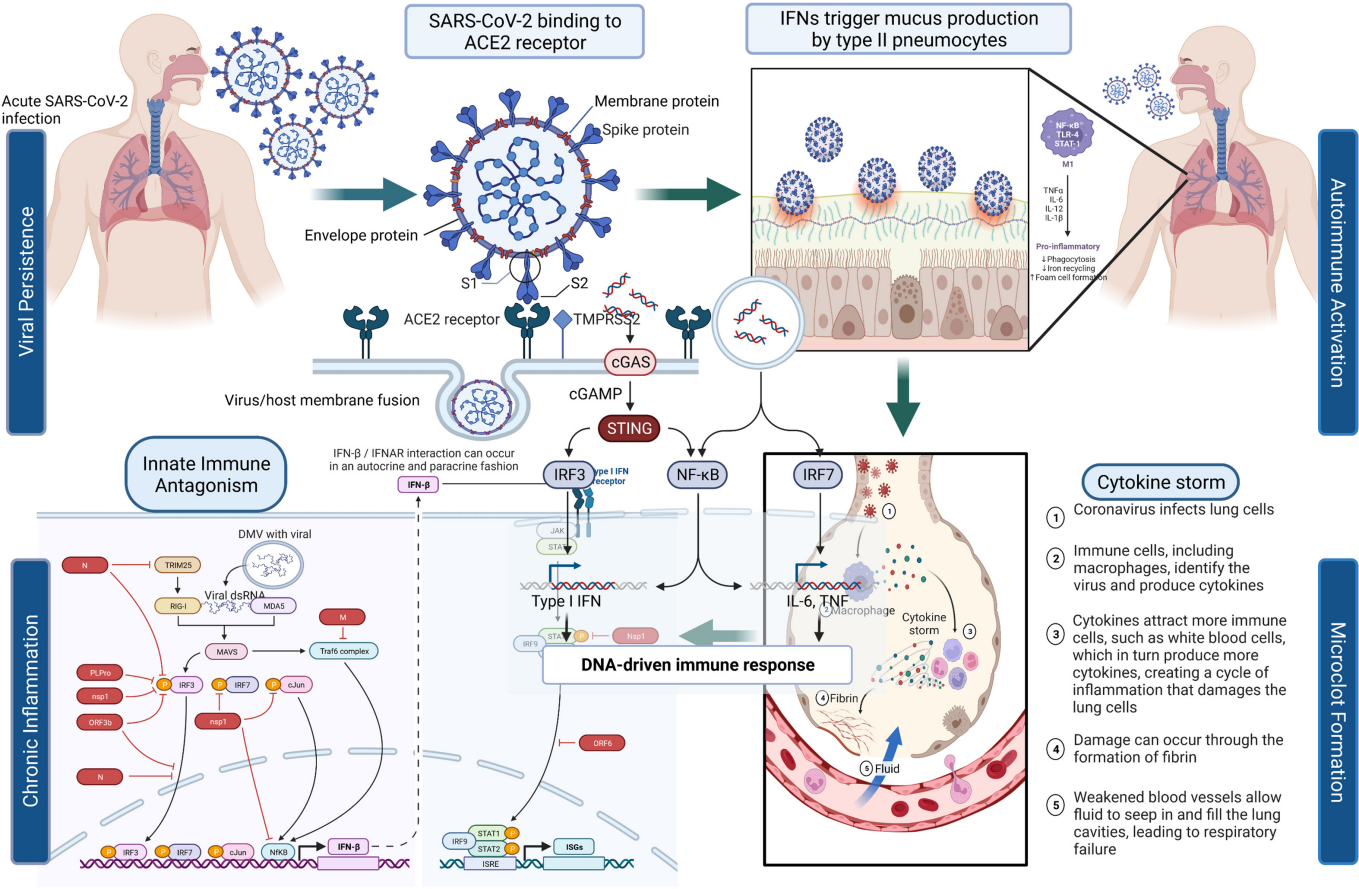
SARS-CoV-2’s entry into the respiratory system and its binding to ACE2 receptor produces a cascade of host responses ranging from chronic inflammation to endothelial dysregulation.

Figure 1 depicts the initial entry of SARS-CoV-2 into the host cell by binding to ACE2 receptor in the respiratory tract and causing disruption of homeostasis by persisting in several organ systems and leading to the eventual development of PASC in a subset of patients. The depiction is for immunological, neurological, and pulmonary dysregulations, which may likely contribute to the hyperactivation of monocyte-derived macrophages in both acute and post-acute phases of the disease. Type I IFN-γ production is delayed leading to increased sensing of microbial threats. This in turn enhances the release of monocyte chemokines by alveolar epithelial cells (pneumocyte types 1 and 2), which direct blood monocytes into the lungs. There are several signaling molecules that induce monocytes to differentiate into pro-inflammatory macrophages via activation of Janus kinase (JAK)–signal transducer and activator of transcription (STAT) pathways. Activated T cells and natural killer cells enhance the recruitment and stimulation of macrophages (derived from monocytes) through the production of granulocyte–macrophage colony-stimulating factor (GM-CSF), IFN-γ, and TNF (see Figure 1).

Alveolar macrophages lead to the activation and release of cytokine and chemokine and aggregation of neutrophils and monocytes that produce toxic mediators resulting in the death of the alveolar cells, fibrin deposition, hyaline membrane formation, the influx of red blood cells, alveolar edema, and dyspnea (Ackermann et al., 2020; Al-Khawaga and Abdelalim, 2020).

Whole-transcriptome sequencing of postmortem lung tissue from COVID-19 patients reveals two distinct molecular signatures: Lung damage caused by a massive metabolic reprogramming due to the upregulation of the unfolded protein response, steroid biosynthesis, and complement activation; and secondarily, ‘cytokine release syndrome’ (CRS) represented by the upregulation of cytokines such as IL-1 and CCL19, but absence of complement activation (Budhraja et al., 2022). Although most patients cleared the viral infection, they succumbed to acute dysbiosis overrepresented by *Staphylococcus cohnii* in ‘classical’ patients and *Pasteurella multocida* in CRS patients (Budhraja et al., 2022).

The association between insulin resistance and lung dysfunction in humans has also been documented in the literature. Another recent study confirmed other previous studies that revealed that the insulin/insulin-like growth factor 1 (IGF) signaling pathway, which plays an important role in energy metabolism, is impaired in organs and tissues of severe COVID-19 patients (Shin et al., 2022). AKT/mTOR/MAPK pathway and ligand/receptor interactions that initiate cellular signaling through IRS/PI3K lie downstream of insulin/IGF signaling pathway but are also additional molecular pathways that are associated with energy uptake and utilization. An impaired IGF pathway engenders insulin resistance or abnormal insulin/IGF response, which also leads to an enhanced metabolic syndrome manifestation such as diabetes, hyperglycemia, hyperlipidemia, and obesity. A COVID-19-impaired insulin/IGF signaling pathway is also associated with the downregulation of various metabolic pathways including the citrate cycle, lipid metabolism, beta-oxidation, amino acid metabolism, carbohydrate metabolism, and respiratory electron transport chain affecting the adipose tissue, liver, lungs, and pancreas resulting in the development of multiorgan dysfunction (Shin et al., 2022).

### Is a persistent virus responsible for the ongoing multi-tissue symptoms?

One of the theories of PASC is viral persistence in the tissues. Therefore, some of the mechanisms that could potentially contribute to PASC’s lingering symptoms are viral-specific pathophysiologic alterations, immunologic aberrations, and inflammation, especially in patients with comorbidities that stimulate excessive cytokine release, lead to destructive immune response and result in autoimmune phenomena and molecular mimicry that causes autoimmunity (Nalbandian et al., 2021). The CRS is a major cause of tissue damage in the pathophysiology of COVID-19 (Ye et al., 2020), is characterized by the activation of innate immunity which along with the epithelial and endothelial cells release several cytokines to block the viral replication. A secondary cytokine cascade is induced downstream by the sustained release of primary cytokines such as IL-6, the most important CRS causative cytokine, or by immune cell signaling (Guo and Thomas, 2017; Ronco and Reis, 2020). In SARS-CoV-2 infection, virus-induced ACE2 downregulation leads to reduced production of Angiotensin 1–7 and accumulation of Angiotensin II, contributing to pulmonary edema, and inflammation (Verdecchia et al., 2020).

## Endocrine system pathophysiology

### What are the potential clinical impacts of SARS-CoV-2 on the endocrine system and the observed symptoms in PASC patients?

There is a large body of research that implicates viral disruption of multiple endocrine glands and their function even though the degree to which endocrine dysfunction contributes to the symptoms experienced by PASC patients remains to be fully elucidated. In a persistent viral environment, the endocrine system not only possesses the requisite ACE2 receptor, but also the TMPRSS2 protein necessary to provide the virion cellular access to dysregulate glandular function that may extend beyond the acute phase of SARS-CoV-2 infection into the Long COVID or post-acute phase. *Can fatigue, the most common reported symptom in PASC, be explained by endocrine dysregulation?* Some of the most frequently reported symptoms of Long COVID such as fatigue, cognitive impairment, and postural hypotension, are also reported by patients with adrenal insufficiency (Kaltsas et al., 2010). However, the fatigue in PASC does not seem to be explained by insufficient adrenal function (Clarke et al., 2022). Moreover, many studies, while offering hypotheses on new-onset diabetes (Gentile et al., 2020) and thyroid disorders (Trimboli et al., 2021) reported endocrine disorders that were unrelated to organ damage.

On the other hand, there is one area that has not received much attention with respect to endocrine-disrupting chemicals (EDCs) such as phthalates, bisphenols, organochlorine pesticides, and perfluorinated alkane substances that degrade the immune system and make children and adults susceptible to suspected hormonal mode of vulnerability (Drucker, 2020) including COVID-19 (Trasande et al., 2016), as supported by experimental studies (Cipelli et al., 2014; Couleau et al., 2015). The investigators conducted biological associations of major EDCs to proteins and diseases known as important COVID-19 comorbidities from the GeneCards and DisGeNET databases, and among the several pathways dysregulated by COVID-19, they found the Th17 and the AGE/RAGE signaling pathways were statistically significant and very auspicious (Wu et al., 2021).

## Hematologic pathologies and their mechanisms

### Are there signaling pathways affected by elevated cytokines in the hematological system?

The elevation of certain cytokines in COVID-19 patients (i.e., IL-6, IL-1β, and IFN-γ), may also be due to the presence of the virus or its remnants in PASC patients. The cytokines can stimulate many processes involved in the activation of immune cells because of changes in the vascular environment, thus promoting greater adhesion and blood procoagulation (Teuwen et al., 2020), and activating dispersed intravascular coagulation and the development of thromboembolic states that can antagonistically affect different tissues, particularly those that are more vulnerable to ischemic processes, such as cardiovascular, cerebrovascular, and pulmonary tissues (Giustino et al., 2020). When the virus binds to the ACE2 receptor and enters the cell, a decreased density of the receptor on the vascular tissue ensues leading to negative regulation of ACE2 activity and subsequent accumulation of Angiotensin II, which causes vasoconstriction, profibrotic, pro-inflammatory effects, as well as tissue fibrosis (Giustino et al., 2020). Therefore, this signaling stimulates immune cell inflammatory processes that can lead to pulmonary degeneration, pulmonary fibrosis, and loss of function accompanied by delayed hypoxemia and anoxia. These cytokines are important activators of the JAK/STAT pathway and NF-κB signaling (Jose and Manuel, 2020). IL-6, which induces the expression of Angiotensin I is itself expressed via JAK/STAT, which creates a progressive feedback loop and results (Jose and Manuel, 2020) in the rapid transmission of extracellular signals from IFNs, cytokines, and hormones, supporting changes in downstream gene expression via STAT-related transcription factors (O’Shea et al., 2015). There are multiple JAKs and STATs that have been noted to impact this pathway, resulting in differential biological outcomes. The cascades of phosphorylation and activation of JAK proteins and the recruitment of STAT proteins, induce STAT dimerization and translocation to the nucleus, where they bind to specific DNA sequences and regulate gene expression (Figure 1; O’Shea et al., 2015).

### Do COVID-19 and Long COVID share signaling pathways that are aberrant?

Some of the potential signaling pathways involved in COVID-19 and PASC pathophysiology involve Toll-like receptors (TLR-3 and TLR-7/8) that recognize SARS-CoV-2 RNA and recruit the inflammatory cascade via type I and II IFN gene expression and NF-κB nuclear translocation (Sabroe et al., 2008; Totura et al., 2015). This stimulates the expression of multiple pro-inflammatory genes including pro-IL-1β, pro-IL-18, TNF, and IL-6 (Li and Verma, 2002; Schultheiss et al., 2022; Tergaonkar et al., 2005). The virus, which may persist as an infectious agent in many different tissues, is also recognized by cytoplasmic NLRP3 that together with ASC and caspase-1 (Casp-1) forms the inflammasome complex leading to the cleavage and release of mature forms of IL-1β and IL-18 (Zhao and Zhao, 2020). The binding of cytokines IL-1β, IL-18, and TNF to specific receptors promotes further NF-κB nuclear translocation and phosphorylation of p38 MAPK. The activation of p38 MAPK leads to further expression of pro-inflammatory cytokines and chemokines (Feng et al., 2019; Grimes and Grimes, 2020). IL-6, an important player in COVID-19, binds IL-6R and gp130 receptors to activate JAK/STAT-3 pathway and then contributes to the CRS observed in COVID-19 (Zhang et al., 2020) and possibly in Long COVID patients. The increased release of inflammatory cytokines IL-1 and IL-6 by the activated M1 phenotype macrophages and the disproportionate activity of Angiotensin II generate endothelial activation, increased permeability, and coexpression of adhesion molecules, helping in the formation of a prothrombotic phenotype (Figure 1; Aird, 2007; Escher et al., 2020). The common hematological abnormalities present mostly in severe COVID-19 patients are lymphopenia, thrombocytopenia, and elevated D-dimer levels (Rahman et al., 2021).

## Dysautonomia in PASC

### What do we know about autonomic dysfunction in PASC?

Although the direct link between COVID-19 and dysautonomia is still theoretical, there may be a connection between neuronal injury of the autonomic pathway and a post-infectious immune-induced mechanism. This neurotrophic virus has multiple indirect routes including the autonomic nervous system by using axonal transport via the olfactory nerve (Li et al., 2020a), via the ACE2 in brainstem and systemic blood circulation (Baig et al., 2020), and via immune injury (Wu and Yang, 2020). The stress induced by the virus may trigger the enhancement of the sympathetic nervous system promoting neuro-hormonal stimulation and activation of pro-inflammatory cytokines (Al-Kuraishy et al., 2021). The typical symptoms experienced by PASC patients such as fatigue, chest pain, palpitations, dyspnea, post-exertional malaise, and ‘brain fog’ are also experienced by patients with orthostatic intolerance and syncope, which suggests the involvement of the autonomic nervous system. Additionally, cardiovascular autonomic dysfunction (CVAD) that appears to be common in PASC is similarly observed in inappropriate sinus tachycardia and POTS. Many POTS patients suffer from fatigue and what is often called ‘brain fog’. Tiny clots (micro-clots) in the brain could explain cognitive ailments arising from clots destroying small fiber nerve cells and driving dysautonomia. It should be emphasized that a growing body of evidence suggests that POTS may be an autoimmune disorder even though POTS is a rare medical condition whose etiology is yet to be established. Its clinical manifestations include the presence of antinuclear antibodies and elevations of ganglionic, adrenergic, and muscarinic acetylcholine receptor antibodies (Gunning et al., 2019). Therefore, the onset of tachycardia following SARS-CoV-2, or any other viral infection can be more frequently explained as a physiological response of a perfectly normal autonomic nervous system. POTS is difficult to identify. It is misdiagnosed in up to 75% of patients and viral infection is recognized as a trigger in up to 41% of cases (Shaw et al., 2019). It has been recently suggested that post-COVID-19 tachycardia syndrome would represent a distinct disease entity, different from classical POTS. There is also autonomic neuropathy that affects the heart, which leads to the proposal that PASC might be the clinical expression of COVID-related CVAD (Bisaccia et al., 2021).

The most common PASC symptom, fatigue, shares some distinct features with CFS. Studies on CFS patients showed impaired cerebral blood flow (van Campen et al., 2020) and reduced heart rate variability (Escorihuela et al., 2020; Nelson et al., 2019), even though these findings could not be confirmed in PASC patients. Furthermore, less than 50% of patients with PASC-related fatigue met the diagnostic criteria for CFS. This suggests that PASC is not just a COVID-19-related manifestation of CFS but a specific pathophysiological entity with a specific CVAD phenotype. But then the follow-up question would be: *When does CVAD occur in COVID-19 patients and how long does it last?*

## Cardiovascular diseases associated with COVID-19—focus on hypertension

Chest pain, dyspnea, and heart palpitations are some of the key symptoms that draw attention to the cardiovascular system (Gluckman et al., 2022). However, cardiovascular disease (CVD) also includes congenital heart disease, coronary artery disease, hypertension (HTN), peripheral artery disease, stroke, and heart failure. Regardless of the severity of COVID-19, many patients have a complicated course of COVID-19 with regard to CVD. This is especially true for viral pneumonia (remembering that COVID-19 is primarily a respiratory infectious disease) that may result in severe systemic inflammation, acute myocardial infarction (MI), acute heart failure, cardiac arrest, venous thromboembolism (VTE), tachyarrhythmias, and stroke (Baldi et al., 2020; Basso et al., 2020; Gao et al., 2020; Lala et al., 2020; Parohan et al., 2020; Shi et al., 2020; Wallentin et al., 2020; Zhou et al., 2020). In a single-centered observational study, up to 329 days following diagnosis with COVID-19, approximately 75% reported persistent cardiac symptoms (Gluckman et al., 2022). In the absence of validated biomarkers for PASC, imaging evidence using cardiac magnetic resonance (CMR) was obtained for myocardial inflammation, pericardial enhancement, and diffuse myocardial edema. Similarly, various abnormalities on CMR as well positron emission tomography have been reported in COVID-19 patients, even in the absence of cardiac symptoms (Hanneman et al., 2022; Puntmann et al., 2020). There are indicators of CVD such as cardiomyocyte injury, quantified by cardiac troponin (cTn) concentrations, heart failure, quantified by N-terminal pro B-type natriuretic peptide (NT-proBNP), and hemodynamic cardiac stress, quantified by natriuretic peptide concentrations that were detected in COVID-19 patients (Flores et al., 2019; Lala et al., 2020; Mueller et al., 2021; Parohan et al., 2020; Shi et al., 2020; Zhou et al., 2020). The potential mechanisms underlying elevations in acute cTn and therefore myocardial injury from non-ischemic causes in COVID-19 patients include direct effect on myocardial cells via ACE2 receptor, cytokine storm, and hypoxia-induced apoptosis as well as myocarditis, Takotsubo syndrome, and pulmonary embolism. The rise in cTn from ischemic causes in COVID-19 patients include type I MI, and type II MI comprising shock, hypoxia, and tachycardia (Mueller et al., 2021).

### As there is a COVID-19-induced diabetes, is there a similar COVID-19-induced CVD like hypertension?

There are two types of HTNs. Essential HTN (aka primary hypertension) is the most common form that is generally idiopathic with undefined mechanisms although it correlates with family history, salt retention, sedentary lifestyle, obesity, smoking, and stress (Carretero and Oparil, 2000). Secondary HTN, on the other hand, is directly linked to comorbid pathophysiological disorders, such as renal, endocrine, and neurological diseases and pregnancy (Rimoldi et al., 2014; Rossier et al., 2017; Tita et al., 2022). Both types of HTN are associated with an increased risk of severe COVID-19 and higher mortality rate in these patients (Lippi et al., 2020). In one study, 31 days after SARS-CoV-2 infection, new-onset HTN (i.e., ≥140 mm Hg systolic BP and/or ≥90 mm Hg diastolic BP) was observed in a small number of patients (Akpek, 2022). Considering that ACE2 plays a negative role in RAAS, a decrease in the ACE2 because of COVID-19, and an increase in the Angiotensin II level, may lead to increase in blood pressure (Akpek, 2022). In a larger control study, 190 patients without prior HTN had higher Angiotensin II level with 8.42% patients recording a rise in blood pressure and a significantly increased level of the cTnI (cardiac troponin I), procalcitonin, and angiotensin (Chen et al., 2021). It is important to note that the cTn concentration in a COVID-19 patient must be seen as the combination of the presence of pre-existing cardiac disease and the acute myocardial injury induced by COVID-19 and its complications (Lala et al., 2020; Mueller et al., 2021). These findings suggest that COVID-19 increases systolic and diastolic BP and may cause new-onset HTN. Altogether, these findings and others regarding cardiovascular symptoms call into question our understanding of viral-mediated myocardial (and pericardial) involvement (Halushka and Vander Heide, 2021; Hanneman et al., 2022; Pellegrini et al., 2021; Puntmann et al., 2020; Webster et al., 2021; Yousif and Premraj, 2022). They additionally raise inquiries about our poor knowledge of the pathophysiology of cardiovascular manifestations and predisposing risk factors associated with Long COVID/PASC whose long-term cardiovascular consequences remain unknown.

## ED related to COVID-19—lesson for PASC

### Does SARS-CoV-2-mediated endothelial damage result in ED that extends to PASC? Could there be endothelial biomarkers in patients with PASC?

A study, which assessed the function of the peripheral endothelia via the reactive hyperemia index (RHI) using peripheral arterial tonometry in PASC patients and age- and sex-matched healthy controls (HCs), showed that there is ample evidence for impaired perfusion and ED (Fluge et al., 2021; Scherbakov et al., 2020). The ED causes inflammation, an intensified immune response, and excessive cytokine release all of which may lead to widespread multiorgan manifestations of the disease (Ostergaard, 2021). It is also noteworthy to mention that the SARS-CoV-2 infection of endothelial cells restructures the cells’ architecture and morphology leading to apoptosis that could persist several weeks after the acute infection. The importance of the endothelium in long-term cardiovascular complications in convalescent patients was underscored by The Working Group on Atherosclerosis and Vascular Biology together with the Council of Basic Cardiovascular Science of the European Society of Cardiology (Evans et al., 2020). In the TUN-EndCOV study, a multivariate analysis showed that ED is an independent risk factor of Long COVID-19 (Charfeddine et al., 2021; Jud et al., 2021).

### Are there biomarkers for endothelial dysregulation?

In a study involving ED and altered endothelial biomarkers in patients with PASC and ME/CFS, 38% of the PASC ME/CFS patients and 31% Long COVID patients (without MS/CFS) showed ED defined by a diminished RHI (<1.67), but none of the HCs exhibited this finding (Haffke et al., 2022). A positive correlation of RHI was found with age, blood pressure, and BMI in PASC but not ME/CFS patients. In a larger cohort of patients and HCs, including post-COVID re-convalescents (PCHCs), Endothelin-1 (ET-1), Angiopoietin-2 (Ang-2), Endocan (ESM-1), IL-8, ACE, and ACE2 were analyzed as endothelial biomarkers. The ET-1 concentration was significantly elevated in both ME/CFS and PASC patients compared to HCs and PCHCs. The serum Angiotensin II concentration was lower in both PCS patients and PCHCs compared to HCs (Haffke et al., 2022). The study authors concluded that a diminished RHI and altered endothelial biomarkers were evidence of ED for a subset of PASC patients suggesting that clinical parameters could be used in association with RHI and varying biomarker profiles to delineate the discrete pathological mechanisms among patient subgroups. In another study designed to understand the role of the ED in COVID-19 and PASC, plasma levels of two proteins released by activated endothelial cells, soluble P-Selectin (sP-Sel) (also released by activated platelets) and von Willebrand factor (VWF) antigen (measures amount of the clotting factor, VWF), and D-dimer (a biomarker of systemic thrombosis), were measured (Osburn et al., 2022). Moreover, these endothelial biomarker levels were compared with the levels of pro-inflammatory cytokine and chemokines, and vascular inflammation biomarkers. The study reports that sP-Sel, VWF, and D-dimer were increased in individuals with COVID-19 pulmonary disease and correlated with proinflammatory cytokines and chemokines, suggesting that COVID-19 is a vascular disease which involves endothelial injury in the context of an inflammatory state (Osburn et al., 2022). A similar study also contends that activated coagulation as quantified by D-dimers is more prominent in COVID-19 as in other pneumonias (Mueller et al., 2021). *How can this information help physicians in selecting patients for the appropriate treatment?* Endothelialitis and VTE play a cardinal role in COVID-19. Consequently, successive measurements of D-dimers may help physicians in the selection of patients for VTE imaging and the administration of the appropriate dosage of anticoagulation for prophylactic or therapeutic purposes.

Mechanistically, the inflammatory mediators, reactive oxygen species, matrix metalloproteases, the glycocalyx, fragments, and the viral proteins may contribute to endothelial glycocalyx damage in COVID-19 (Zha et al., 2022). Heparin sulfate (HS) has been shown to regulate the activation of the bradykinin pathway, which is involved in local inflammation and vascular permeability (Buijsers et al., 2020). Elevated plasma heparanase activity in COVID-19 patients can lead to the activation of bradykinin pathway by the cleaving of HS and can subsequently trigger the inflammatory response and vascular leakage. Other potential mechanisms that account for coagulopathy in COVID-19 and cause endothelial function as well as cardiovascular disorders include plasmin-mediated increased binding of SARS-CoV-2 to ACE2 receptors, elevated levels of fibrinogen and an unnecessary fibrin polymerization, cytokine-mediated disseminated intravascular coagulation, activation of thrombin and suppression of fibrinolysis by plasminogen activators and PAI-1 inhibitors in ARDS, inhibition of plasmin by antiplasmins, and direct viral infection/endotheliitis (Mueller et al., 2021). All these demonstrations of endothelial dysregulation have convinced some scientists to consider COVID-19 not just a respiratory disease, but a vascular one as well. The rationale is that ACE2 is the doorway for SARS-CoV-2 to bind to and infect cells, and COVID-19 is associated with several acute clotting syndromes that precipitate debilitating symptoms in PASC patients.

## Potential mechanisms for neurocognitive impairment in PASC

SARS-CoV-2 can infect the brain, causing neuroinflammation (Pan et al., 2020). Moreover, inflammation elsewhere in the body can activate the innate immune system (Figure 1) in the brain via both humoral and retrograde neural signals, largely involving the vagus nerve (Poon et al., 2015; VanElzakker, 2013). Different study model systems such as autopsies, animals and organoids used to study the infective potential of the SARS-CoV-2 virus show that SARS-CoV-2 can reach and infect neurons and cells of the CNS and produce neuroinflammation (Matschke et al., 2020; Song et al., 2020). Neuropsychological symptoms are frequent in PASC survivors of COVID-19 who often experience lingering neurological symptoms analogous to cancer-therapy-related cognitive impairment, which involves white matter microglial reactivity and consequent neural dysregulation. SARS-CoV-2 infection has been shown to affect white-matter-selective microglial reactivity in mice and humans by impairing hippocampal neurogenesis, decreasing oligodendrocytes, and promoting myelin loss with a concomitant increase in CSF cytokines/chemokines including CCL11 (Fernández-Castañeda et al., 2022a). Accordingly, cognitive impairment manifested even with mild COVID symptoms parallel the neuropathophysiology that is observed in people with cancer therapy (Fernández-Castañeda et al., 2022b). The question remains: *How does the virus manage to get to the brain?* One potential pathway to reach the CNS is via hematogenous dissemination from severely infected upper and lower respiratory system by way of systemic inflammation that raises the permeability of blood–brain barrier (BBB) and permit circulating non-BBB-crossing molecules to adversely affect brain function (Proal and VanElzakker, 2021). More importantly for PASC, during acute infection this porousness can also allow for viral neuroinvasion that may persist past the acute infection phase. Although the mechanism is not well known, this neuroinvasion can occur directly or indirectly through host immune cells infected with the virus and are actively transported into the CNS (Dando et al., 2014; Lauer et al., 2018). A large MRI registry study that examined changes in the brain before and a mean of 141 days following SAR-CoV-2 identified reductions global brain size and gray matter thickness in orbitofrontal cortex/parahippocampal gyrus. Importantly, the study also identified increased markers of tissue damage in regions associated with the primary olfactory cortex following infection. These data suggest that COVID-19 has profound impact on the brain, especially the olfactory cortex, which may explain changes of taste and smell and may indicate CNS infection from direct extension from the olfactory system (Douaud et al., 2022).

### What could explain neurological symptoms in PASC patients?

Concerning pathophysiology, virus neurotropism leading to sustained neuroinflammation of central and peripheral nervous systems could explain neurocognitive impairment or symptoms of mental health disorders, as has been proposed by numerous studies detailed in a review (Castanares-Zapatero et al., 2022). It is also likely that central, peripheral, psychological, and physiological factors play a role in the development of PASC fatigue. A chronicle of Long COVID describes that congestion of the glymphatic system and the subsequent toxic build-up within the CNS, caused by an increased resistance to cerebrospinal fluid drainage through the cribriform plate because of olfactory neuron damage, may contribute to PASC fatigue (Wostyn, 2021). Emotional and cognitive disturbances could also be partially traced back to metabolic changes linked to tissue damage, repair, and immune function in multiple areas of the nervous system (Holmes et al., 2021). To support this hypothesis, use of animal models of COVID has proven valuable. In the golden hamster model of SARS-CoV-2 infection, sustained inflammatory pathology correlated with behavioral abnormalities not found in influenza A virus infection (Frere et al., 2022). Exclusive features included anosmia and anxiety-like behaviors, a unique neural transcriptional profile, and a persistent neuroimmune response reminiscent to what has been observed in humans (Frere et al., 2022). In this study, differential gene expression data revealed significant enrichments for metabolic, synaptic signaling, neural plasticity, and immune-related pathways. These data provide validity for modeling and dissecting the physiopathology of neuropsychiatric symptoms of PASC. In addition, these results set a benchmark for comparison between PASC and other clinical conditions. Other neuropsychological symptoms of PASC such as ME/CFS, and ‘brain fog’ could be explained by direct integration of viral genome into mitochondrial DNA, impairing energy metabolism, and oxygen availability and utilization (Wu et al., 2021). Metabolic fitness can also be altered by impaired autophagy, which relates to inadequate clearance of debris by microglia (Awogbindin et al., 2021). As an indirect effect, it has been proposed that impaired pulmonary function leads to decreased systemic oxygenation, including the CNS. Alternatively, when lingering, systemic inflammation may disrupt the brain–blood barrier, cause inflammatory damage, and neurocognitive deficit (Stefano et al., 2022). SARS-CoV-2 may also enter taste buds through ACE2 protein found in some taste receptor cells, causing taste changes, and disrupted stem cells. Further research is needed to understand this process (Doyle et al., 2021). Though there are several hypotheses, the exact mechanisms, and the attempt to identity a unifying pathophysiological cause (e.g., neuroinflammation) of many of the described symptoms of PASC remain elusive (Tate et al., 2022). Progress in this direction is needed as the neuropsychiatric manifestations of PASC may increase the risk long-term of neurocognitive diseases (e.g., dementia) with common mechanistic features (Liu et al., 2022).

## GI effects of COVID-19 and Long COVID

The extrapulmonary disease that SARS-CoV-2 inflicts upon the body also extends to the GI tract where there is evidence that the virus may persist where the ACE2 receptor is highly expressed (Settanni et al., 2021). Given that the severity of COVID-19 is typically engendered by a set of comorbidities such as HTN, diabetes, obesity, and/or advanced age, the GI symptoms, such as diarrhea, vomiting, or abdominal pain during the early phases of the disease can be debilitating (Lamers et al., 2020). Symptoms can last for years as is the case for post-infectious irritable bowel syndrome (PI-IBS), which is punctuated by the persistence of abdominal discomfort, bloating and diarrhea that continue despite clearance of the inciting pathogen, usually bacterial origin (Dunlop et al., 2003; Kim et al., 2006; Settanni et al., 2021). Again, the culprit in all these acute and post-acute COVID-19 symptoms including the impairment of bowel physiology may be a dysregulation of ACE2-mediated functions due to a competitive mechanism of the virus on ACE2 receptor or from a downregulation of its anti-inflammatory activity. To date, there is a dearth of data on the GI sequelae of SARS-CoV-2 infection. This may be due to the uncertainty over COVID-19’s path of action on the gut physiology.

### Is it viral persistence or its aftereffects?

It is conceivable that recovery from COVID-19 may not absolve the GI tract of functional bowel diseases that may precipitate due to potential pathophysiological alterations such as disruption of the intestinal barrier, dysbiosis, mucosal microinflammation, post-infectious states, immune dysregulation, and psychological stress (Settanni et al., 2021). The undermining of the intestinal homeostasis by the virus not only may perturb the microbial composition but also increase inflammatory cytokines. For example, SARS-CoV-2 infection has been shown to lead to persistent altered gut mucosal integrity and microbial translocation resulting in increased NF-κB signaling and generalized inflammation in those with PASC (Giron et al., 2022). Inflammation can be potentially more harmful to the gut than the virus itself. The virus initiates a cascade of immune reactions that are self-activating via autoantibodies with the host cytokine pathways triggering adverse microbiota interactions in a continuous and circuitous manner. For this reason, it is crucial to investigate how intestinal bacteria respond to SARS-CoV-2 infection and the effect of their metabolic products to help determine novel biomarkers of the disease and its long-term impact on the GI tract to enable new therapeutic targets.

## Potential biological underpinnings of pediatric PASC

While much of our current understanding of PASC comes from adults, one of the first PASC phenotypes occurred in children. Shortly after the emergence of SARS-CoV-2, rare cases of multisystem inflammatory syndrome in children (MIS-C) were documented (Riphagen et al., 2020). It is now understood that MIS-C occurs weeks after SARS-CoV-2 infection and is characterized by persistent fevers, massive inflammation, and multiorgan involvement including long-term neurologic complications. The most striking clinical manifestation is profound myocardial dysfunction resulting in shock; however, extracardiac organ system dysfunction is also common, particularly GI (Cheung et al., 2020; Feldstein et al., 2020; Riphagen et al., 2020). The mechanisms which underlie MIS-C are not fully understood but emerging evidence points toward immune system dysfunction. Acute MIS-C is defined by massive elevations in inflammatory cytokines, particularly those related to myeloid recruitment and mucosal immunity (Consiglio et al., 2020; Gruber et al., 2020). The production of autoreactive antibodies is also characteristic of acute MIS-C with antibodies specific for endothelial proteins detected in many patients (Consiglio et al., 2020; Gruber et al., 2020; Ramaswamy et al., 2021). Aberrant activation of T cells is a consistent finding as well (Vella et al., 2021), with a potentially pathognomonic role for biased expansions of T cell clones (Porritt et al., 2021). Activated T lymphocyte cell expansions resemble those seen following bacterial superantigen stimulation with additional evidence suggesting that motifs from the SARS-CoV-2 spike protein may be capable of promoting autoimmunity (Noval Rivas et al., 2022). These immune derangements along with elevated biomarkers suggesting mucosal barrier breakdown have been linked to detectable antigenic persistence in circulation (Yonker et al., 2021), suggesting an additional role for viral persistence in the pathogenesis of MIS-C. Treatment for MIS-C has included steroids, intravenous immunoglobulin, and monoclonal antibodies targeting IL-6 and IL-1Ra (Feldstein et al., 2020). Fortunately, following treatment for MIS-C, short to mid-range reported health outcomes have been excellent (Farooqi et al., 2021; Matsubara et al., 2022). Pediatric PASC outside of MIS-C has been more difficult to quantify, with estimates ranging from 4% to 25% of previously infected children experiencing PASC symptoms (Izquierdo-Pujol et al., 2022). Risk factors for development of pediatric PASC include severity of acute infection, younger age (<5 years), and presence of chronic health conditions (Rao et al., 2022). In case reports or small series, endothelial damage and encephalomyelitis have been identified in children with PASC (Buonsenso et al., 2021; Lindan et al., 2021) but underlying mechanisms remain undefined. Based on our current knowledge of MIS-C and the evidence that pediatric immune responses to SARS-CoV-2 are distinct from adults (Pierce et al., 2020; Weisberg et al., 2021; Yoshida et al., 2022), understanding immune responses in pediatric PASC must be prioritized. To better characterize pediatric PASC subtypes and define the mechanisms giving rise to persistent symptomatology, awareness, and identification followed by study enrollment of relatively rare cases is critical to maximize our ability to understand and treat this emerging pediatric health threat.

## Unanswered questions about PASC

What are PASC factors? When can the factors be assayed in the course of disease progression? Are these factors interrelated or independent? What are the immune and genomic signatures of PASC? What is the eventual public disease burden of PASC? What does PASC mean for patients and what can be done about it? The unknowns about PASC are too numerous to list. We still do not know if there is a correlation between PASC and the duration/frequency/severity of the initial SARS-CoV-2 infection. Also, the subsequent iterations of the virus (i.e., omicron variants) with factors like age, gender, demographics, and comorbidities are not clear. The longitudinal collection of biospecimens and deep phenotyping of PASC subjects in the RECOVER study will provide a rich set of resources to answer key prognostic, epidemiologic and pathophysiologic questions about PASC and will serve as a platform for testing interventions to prevent or treat PASC.

We urgently need comprehensive epidemiologic studies via surveys/questionnaires and by interrogating the electronic health record databases and through longitudinal study enrollment that allow for a more complete understanding of the prevalence ad incidence of PASC within various populations. Furthermore, comprehensive examination of blood and tissues using synergistic methods such as proteomics, metabolomics, transcriptomics, viral nucleic acid and protein testing, and virus-specific and auto-reactive antibody characterization will be needed. Additionally, comprehensive immunophenotyping of PASC can be performed to identify cell markers and to provide further pathophysiologic understanding of the interplay between immune responses, inflammation, and PASC phenotypes. It must also be noted that the mechanisms underlying Long COVID/PASC may not be uniform across all individuals. Among the more prominent sociodemographic and clinical risk factors, the severity of acute COVID-19 infection, female sex, advanced age, pre-existing diabetes mellitus, more than five early symptoms, early dyspnea, and prior psychiatric disorders have been identified (Carfì et al., 2020; Subramanian et al., 2022; Yong, 2021). Additional variables that need to be considered include the variant of SARS-CoV-2 infection and those who develop PASC and the role of vaccination and antiviral therapy in acute infection (Antonelli et al., 2022; Ledford, 2022). Double-vaccinated participants with COVID-19 caused by the Omicron BA.1, BA.4, and BA.5 variants were less likely to develop PASC, as compared to those infected by the Delta variant. It is also likely that most of the symptoms and pathologies associated with COVID-19 persist in Long COVID perhaps because of the spike protein, which is also the target of the vaccination. The currently known and suspected mechanisms and molecular features for COVID and potentially for Long COVID/PASC pathologies are shown in Table 1.

**Table 1.** Proposed molecular, pathobiological, and pathophysiological mechanisms related to Long COVID symptoms.

Dysregulated system	Mechanism	Biological/molecular factor*	Signaling pathway	Reference
*Autonomic*	Autoimmune dysfunction of the autonomic nervous system; cardiovascular autonomic dysfunction (CVAD); post-intensive care syndrome (PICS); orthostatic intolerance (OI); syncope; postural orthostatic tachycardia syndrome (POTS)-like dysfunction; (ME/CFS)-like dysfunction; antinuclear antibodies	**↑**Ganglionic, adrenergic, and muscarinic acetylcholine receptor antibodies **↑**G-protein-coupled receptor autoantibodies	Sympathetic nervous system; cytokine storms; activation of renin–angiotensin system	Al-Kuraishy et al., 2021; Baig et al., 2020; Bisaccia et al., 2021; Escorihuela et al., 2020; Gunning et al., 2019; Li et al., 2020a; Nelson et al., 2019; Shaw et al., 2019; van Campen et al., 2020; Wu and Yang, 2020;
*Cardiovascular*	Myocardial hypertrophy; focal myocardial fibrosis; acute myocardial infarction; cardiac hypertrophy; endothelial dysfunction and coagulation activation; neutrophil extracellular traps (NETs); inflammation-coagulation (Factor XII); direct complement activation (inflammation)	**↑**Thrombin **↓**Plasmin **↓**Fibrinolysis **↑** NT-proBNP **↑**cTnI **↑**Serum lactate dehydrogenase **↑**Creatine kinase	Aryl hydrocarbon receptor (AhR) signaling through an IDO-Kyn-dependent pathway (hypoxia induction)	Baldi et al., 2020; Basso et al., 2020; Flores et al., 2019; Gao et al., 2020; Hanneman et al., 2022; Lala et al., 2020; Liu et al., 2020b; Mueller et al., 2021; Parohan et al., 2020; Puntmann et al., 2020; Shi et al., 2020; Wallentin et al., 2020
*Endocrine*	Damage to thyroid gland; thyroiditis/thyrotoxicosis	**↓**Thyroxin-3, **↑**ACE2, **↑**TMPRSS2	The Th17 and the AGE/RAGE signaling pathways	Clarke et al., 2022; Lazartigues et al., 2020; Wei et al., 2007; Wu et al., 2021
*Endothelial*	Endothelialitis; thromboembolism; endothelial glycocalyx damage	**↑**sP-Sel, vWF, and D-dimer **↑** Endothelin-1 (ET-1) **↓** Angiotensin II **↑**Proinflammatory cytokines and chemokines **↑**Plasma heparinase	Bradykinin pathway	Buijsers et al., 2020; Flores et al., 2019; Mueller et al., 2021; Osburn et al., 2022; Scherbakov et al., 2020; Zha et al., 2022
*Gastrointestinal*	Impaired barrier function; gut inflammation, altered serotonin metabolism and gut microbiota dysbiosis, *Ruminococcus gnavus* positively correlated with inflammatory markers; Clostridia was negatively correlated, fungal translocation leading to NF-κB activation and inflammation	**↑** Ruminococcus gnavus, **↑**Fecal calprotectin **↑**Plasma serotonin (5-hydroxytrytamine, 5-HT)	Interferon gamma (IFN*-*γ) signaling	Chandrashekhar Joshi and Pozzilli, 2022; Giron et al., 2022; Schultheiss et al., 2022
*Hematological*	Vascular dysregulation; hypercoagulopathy; endothelialitis; hemostatic changes that leave the endothelium inflamed, pre-adhesive, and prothrombotic; thrombocytopenia, deep vein thrombosis	**↑**Plasminogen activator inhibitor factor I (PAI) **↑**Tissue factor (TF) **↑**von Willebrand factor (vWF) **↑**D-dimer **↓**Hb **↑**GM-CSF	p38 MAPK; Antibody-dependent cellular toxicity	Aird, 2007; Boisramé-Helms et al., 2013; Giustino et al., 2020; Hamming et al., 2004; Hamming et al., 2007; Hussman, 2020; Liu et al., 2020a; Rahman et al., 2021; Riphagen et al., 2020; Zachariah et al., 2020
*Immunological*	Dysregulated and heightened immune system; prolonged inflammation leading to multiorgan dysfunction; cytokine release syndrome (CRS); Mast Cell Activation Syndrome (MCAS); neutrophil extracellular traps (NETs); pattern recognition receptors (PRRs) including Toll-like receptor (TLR); retinoic acid-inducible gene-I (RIG-I)-like receptors; oxidative stress	**↑**IL-1β, IL-2, IL-6 **↑**IL- 8, IL-10 **↑**IL-IL-12, IL-17 **↑**IL-18 **↑**IFN-γ, NF-κB; **↑**IFN-I and IFN-III **↑**TNF, **↑**CXCL-10, **↑**p38 MAPK **↑**JAKs and STATs **↑**GM-CSF **↑**TGF- β **↑**ESR	JAK/STAT; Toll-like receptor pathways (TLR-3 and TLR 7/8); AKT/mTOR/MAPK pathway; FcyRIIA; NF-κB p65 and p38 MAPK, 1/2 (STAT1/2) pathway	Blanco-Melo et al., 2020; Feng et al., 2019; Guo and Thomas, 2017; Jose and Manuel, 2020; Li and Verma, 2002; O’Shea et al., 2015; Pablos et al., 2020; Ronco and Reis, 2020; Tarentino and Maley, 1975; Tay et al., 2020; Tergaonkar et al., 2005; Totura et al., 2015; Wirth and Scheibenbogen, 2021; Ye et al., 2020; Zhang et al., 2020; Zhao and Zhao, 2020
*Neurological*	Neuro-inflammation; coagulopathy; micro-thrombosis; aberrant immune response; metabolic brain disorders; stroke; Guillain–Barré syndrome; hemodynamic and coagulation disorders; arrhythmia; nervous system damage	**↑**IL-18, IL-6, and IL-8 **↑**CCL-11 **↑**Neurofilament light (NFL) **↑**Glial fibrillary acidic protein (GFAP)	Humoral and retrograde neural signals; blood–brain barrier (BBB)	Awogbindin et al., 2021; Castanares-Zapatero et al., 2022; Dando et al., 2014; Fernández-Castañeda et al., 2022a; Fernández-Castañeda et al., 2022b; Frere et al., 2022; Holmes et al., 2021; Lauer et al., 2018; Matschke et al., 2020; Pan et al., 2020; Poon et al., 2015; Proal and VanElzakker, 2021; Sahin et al., 2022; Song et al., 2020 ; Tate et al., 2022; VanElzakker, 2013; Wostyn, 2021; Wu et al., 2020
*Pancreatic*	Direct insulin resistance and beta cell damage; indirect autoimmunity inflammation; stress and steroid-induced hyperglycemia	**↑**PKR, PERK, ISR via viral RNA and Cytokine storm, IRS **↑**IL-6 and TNF-α **↓**REST (RE1-silencing transcription factor) **↑**Amylase and lipase **↑**HLA **↑**Propionic acid **↑**Isobutyric acid	Insulin signaling pathway, ISR	Shin et al., 2022
*Renal*	Glomerulopathy; microangiopathy	**↑**ACE2 receptor, **↑**IFN-I and IFN-III	Renin–angiotensin–aldosterone system (RAAS) disorders	Blanco-Melo et al., 2020; Hamming et al., 2004; Harmer et al., 2002; Nalbandian et al., 2021 Tay et al., 2020; Zou et al., 2020
*Respiratory*	Pulmonary fibrosis; pulmonary thromboembolism; pneumonia; pulmonary vascular damage; cytokine release syndrome; dysbiosis (increased *Staphylococcus cohnii* and *Pasteurella multocida*); lipid metabolism; beta-oxidation; amino acid metabolism; and carbohydrate metabolism; hyperactivated T-lymphocytes and inflammatory macrophages; NETosis	**↑**TNF-α, IL-1 **↑**IL-IL-1β, IL-6 **↑**IL-12 **↑**MCP-1 **↑**GM-CSF **↑**CCL19 **↑**CCR6 + Th17 **↑**IL-8 (CXCL8)	Insulin/IGF signaling pathway Citrate cycle; respiratory electron transport chain; Th17 cell differentiation pathway	Ackermann et al., 2020; Budhraja et al., 2022; D’Errico et al., 2020; George et al., 2020; Griffin et al., 2020; Grosse et al., 2020; Jin et al., 2021; Jonigk et al., 2022; Kosuge et al., 2012; Medina-Enríquez et al., 2020; Morganstein et al., 2021; Sakr et al., 2020; Shin et al., 2022; Sidarta-Oliveira et al., 2020; Verdecchia et al., 2020; Xie et al., 2021; Yaméogo et al., 2011; Ye et al., 2020
*Multisystem Inflammatory Syndrome in Children (MIS-C*)	Kawasaki disease; cardiac involvement with macrophage activation; persistent T-cell immune response; mucosal barrier breakdown	**↑**IL-6 and IL-1Ra	???	Feldstein et al., 2020; Sadanandam et al., 2020

* **↑** and **↓** arrows indicate increased and decreased levels, respectively, of biological and/or molecular factors.

## Prevailing hypotheses for PASC

There are various hypotheses underlying Long COVID/PASC symptoms. The first points to the immune system going haywire after the initial virus infection, triggering a long-lasting inflammatory response that inflicts havoc on multiple organ systems. The second hypothesis is that this long-lasting inflammation may be due to the continued presence of virus or viral particles in organs such as the gut but may also be due to the reactivation of latent pathogens such as EBV, the most common cause of mononucleosis. The third hypothesis states that COVID-19 elicits a radical response analogous to an autoimmune disease that causes antibodies to target and destroy the body’s own tissues. The fourth leading hypothesis stipulates the formation of microclots, tiny blood clots that cause coagulopathy and lead to thrombosis. Therefore, the conclusion from this review on tissue damage is that there may be more than a single mechanism in motion for Long COVID (PASC) patients who experience a constellation of symptoms. Certain mechanisms may dominate over others in individual patients. There may be potential biomarkers or signaling pathways for different tissues or organs affected as well as therapeutic applications mostly in the form of inhibitors of the presumptive biomarkers of various tissues presented in Table 1, that could be employed for PASC sufferers until the underlying individual or collective mechanisms giving rise to their symptoms are clearly delineated. Furthermore, there is an urgent need to design experimental protocols that can distinctively identify the underlying mechanisms or pathways that are responsible for the clinical manifestations exhibited by Long COVID or PASC patients.

## Authorship

Authorship has been determined according to ICMJE recommendations.
